# Supplementary material for: Characterizing cerebral metabolite profiles in anorexia and bulimia nervosa and their associations with habitual behavior
Source: Transl Psychiatry. 2022 Mar 15;12:103. doi: 10.1038/s41398-022-01872-7 (PMC8924163; doi:10.1038/s41398-022-01872-7)
Supplement: Supplementary file 1 — Supplemental Material [file 41398_2022_1872_MOESM1_ESM.docx]

**Supplementary Material: Characterizing cerebral metabolite profiles in anorexia and bulimia nervosa and their associations with habitual behavior**

Margaret L. Westwater, Alexander G. Murley, Kelly M.J. Diederen, T. Adrian Carpenter, Hisham Ziauddeen & Paul C. Fletcher

1. **Methods**

**Study procedures**

As described in the main text, potential volunteers completed an outpatient screening session at Addenbrooke’s hospital (Cambridge, UK), which included blood sampling, body composition testing, cognitive testing and two validated clinical interviews to ascertain diagnostic information. Twelve patient participants who lived outside of Cambridgeshire completed a remote screening session to reduce participant burden. These individuals completed all blood sampling and anthropometric procedures during the inpatient study session. Finally, among other exclusion criteria, individuals with obesity (BMI > 29.9 kg/m^2^) were not eligible to take part. This exclusion criterion was related to a complementary arm of the study, which examined endocrine and gut hormone responses to acute stress(1), and these hormonal responses may be further altered in obesity. Eligible volunteers were asked to perform at-home saliva sampling on two days prior to returning for their inpatient testing session.

Participants were admitted to Addenbrooke’s hospital for their two-day inpatient testing session at either 8.00 or 9.00 AM. After measuring the participant’s height and weight, they were offered a standardized breakfast that was followed by a cognitive testing battery and a mid-morning snack. Then, participants began a 6-hour fasting period. A cannula was placed ~1 hour prior to MRI scanning to facilitate serial blood sampling of cortisol and gut hormones (1). MRI scans began between 1.30 and 2.30 PM to account for diurnal fluctuations in cortisol. During the scan, participants performed an inhibitory control task twice: immediately before and after either a stress induction or a control task on each day (3). The order of inductions was randomized across participants. ^1^H-MRS data were acquired prior to fMRI scanning on the ‘control’ day. Following the scan, participants had an unsupervised free-choice meal; individuals who did not consume their estimated energy requirements during the meal were offered an evening snack at 7.30 PM. On day 2, participants repeated the protocol, and they were debriefed and discharged following the free-choice meal.

**Data analysis – Behavior**

Performance on the instrumental learning task [accuracy, reaction time (RT)] was evaluated using linear-mixed effects models (LMMs) and multiple regression. Outlier RT values were defined as those >1.5 times the interquartile range above the 75^th^ percentile and below the 25^th^ percentile, and for the instrumental learning stage, outliers were evaluated for each task block. A rank-based inverse normal transformation was applied to the remaining RTs to minimize positive skew when necessary [R package ‘RNOmni’ (2)].

Each model included fixed effects of group (AN-BP>HC, BN>HC) and task condition (block for the instrumental learning stage; stimulus or outcome value for discrimination and slips-of-action stages, respectively), where random intercepts for task condition were nested within the random effect of the subject. Group differences were evaluated using non-orthogonal contrast coding, in which AN-BP and BN groups were compared to controls (e.g., treatment contrasts). Finally, multiple regression was used to assess group differences in sensitivity to outcome devaluation (stage 2) and mean differences in the proportion of responses to valued and devalued outcomes.

**Data analysis – Neuroimaging**

***Data acquisition and pre-processing***

Spectra were collected using a semiLASER (sLASER) sequence, which was selected over a vendor-provided PRESS protocol due to its improved localization, spectral quality and replicability (4, 5). Specifically, previous sLASER experiments have obtained highly reproducible neurochemical profiles for five major metabolites, including the three metabolites of interest in the present study (i.e., glutamate, NAA, *myo*-inositol), where coefficients of variance were <5% at both 3 and 7 Tesla (6).

Pre-processing of ^1^H-MRS spectra was performed using the MRSpa MATLAB package (<https://www.cmrr.umn.edu/downloads/mrspa/>). Raw MRS free induction decays (FIDs) were corrected for eddy current effects, and a zero-order phase correction was applied. FIDs were corrected for temporal (or “frequency”) and phase drifts using the cross-correlation method, which minimizes the frequency and phase difference, respectively, between single-shot MRS data. Correction of frequency and phase drifts served to mitigate broadening of the spectra, improve the signal-to-noise ratio and prevent line shape distortion (7). Finally, FIDs were then visually inspected for outlier transients (e.g., those containing a large residual water peak or noise-only spectrum) as these would be indicative of large motion artifacts. Outlier FIDs (<10%) were removed from 26 ilPFC and 18 occipital spectra before summation.

***Voxel segmentation***

To control for partial volume effects on metabolic estimation, we calculated the proportion of grey matter (GM), white matter (WM) and cerebrospinal fluid (CSF) in each voxel of interest (VOI). Whole-brain, voxel-based tissue segmentation of the T1-weighted anatomical scan was conducted in SPM12 (Wellcome Department of Clinical Neurology, London), using ICBM Tissue Probabilistic Atlases. Each VOI was then coregistered to the anatomical image, and the percentage of GM, WM and CSF tissue within the VOIs was computed using both the GM and WM probability maps, using custom Matlab code provided by Dr Dinesh Deelchand of the University of Minnesota.

***Exploratory correlation analyses between cerebral metabolites and eating disorder symptom severity***

While our primary analyses examined group differences between patient participants and controls, we conducted a complementary set of exploratory correlation analyses to determine if regional glutamate, *myo*-inositol and NAA concentrations were associated with BMI, eating disorder symptom severity [total scores on the Eating Disorder Examination Questionnaire; EDEQ (8)] and depressive symptoms [Beck Depression Inventory-II; BDI-II (9)]. Pearson correlation coefficients were computed across the full sample and within each participant group, and results were FDR-corrected for multiple comparisons.

1. **Results**

**Behavior - Explicit knowledge of stimulus, response and outcome associations**

Upon completion of the instrumental learning task, participants responded to a set of questions that indexed their explicit knowledge of stimulus, response and outcome associations. Questionnaire data were available from 80 participants (n=20 AN-BP, n=32 BN, n=28 HC), and knowledge of stimulus-outcome pairings and correct responses did not differ between groups (all p’s > .05; see Figure S1).

**References**

1. Westwater ML, Mancini F, Shapleske J, Serfontein J, Ernst M, Ziauddeen H, Fletcher PC (2020): Dissociable hormonal profiles for psychopathology and stress in anorexia and bulimia nervosa. *Psychol Med*. 1–11.

2. McCaw Z (2019): RNOmni: Rank Normal Transformation Omnibus Test. Retrieved from https://cran.r-project.org/web/packages/RNOmni.

3. Westwater ML, Mancini F, Gorka AX, Shapleske J, Serfontein J, Grillon C, *et al.* (2021): Prefrontal responses during proactive and reactive inhibition are differentially impacted by stress in anorexia and bulimia nervosa. *J Neurosci*. doi: 10.1523/jneurosci.2853-20.2021.

4. Deelchand DK, Adanyeguh IM, Emir UE, Nguyen TM, Valabregue R, Henry PG, *et al.* (2015): Two-site reproducibility of cerebellar and brainstem neurochemical profiles with short-echo, single-voxel MRS at 3T. *Magn Reson Med*. 73: 1718–1725.

5. Deelchand DK, Kantarci K, Öz G (2018): Improved localization, spectral quality, and repeatability with advanced MRS methodology in the clinical setting. *Magn Reson Med*. 79: 1241–1250.

6. Terpstra M, Cheong I, Lyu T, Deelchand DK, Emir UE, Bednařík P, *et al.* (2016): Test-retest reproducibility of neurochemical profiles with short-echo, single-voxel MR spectroscopy at 3T and 7T. *Magn Reson Med*. 76: 1083–1091.

7. Near J, Harris AD, Juchem C, Kreis R, Marjańska M, Öz G, *et al.* (2020): Preprocessing, analysis and quantification in single‐voxel magnetic resonance spectroscopy: experts’ consensus recommendations. *NMR Biomed*. e4257.

8. Fairburn CG, Beglin SJ (1994): Assessment of eating disorders: Interview or self‐report questionnaire? *Int J Eat Disord*. 16: 363–370.

9. Beck AT, Steer RA, Ball R, Ranieri W (1996): Comparison of Beck Depression Inventories -IA and -II in psychiatric outpatients. *J Pers Assess*. 67: 588–97.

**Table S1**. Spectral quality metrics

| **Characteristic** | AN-BP  M(SD) | BN  M(SD) | HC  M(SD) | *F* statistic | *P*-value |
| --- | --- | --- | --- | --- | --- |
| Water line width (Hz) |  |  |  |  |  |
| *Right inferior frontal cortex* | 8.35 (0.58) | 8.54 (0.78) | 8.56 (0.83) | 0.56 | .57 |
| *Right occipital cortex* | 7.63 (0.31) | 7.76 (0.45) | 7.82 (0.44) | 1.25 | .29 |
| Sign-to-noise-ratio |  |  |  |  |  |
| *Right inferior frontal cortex* | 49.73 (7.23) | 50.66 (7.43) | 51.7 (8.32) | 0.42 | .66 |
| *Right occipital cortex* | 51.64 (11.84) | 50.78 (11.71) | 54.60 (12.58) | 0.83 | .44 |
| Glutamate CRLB |  |  |  |  |  |
| *Right inferior frontal cortex* | 4.22 (0.75) | 4.22 (0.79) | 4.23 (0.86) | .003 | .99 |
| *Right occipital cortex* | 5.64 (2.34) | 5.45 (2.01) | 5.00 (1.20) | 0.83 | .44 |
| Myo-inositol CRLB |  |  |  |  |  |
| *Right inferior frontal cortex* | 4.32 (1.09) | 4.03 (0.78) | 3.80 (0.61) | 2.53 | .09 |
| *Right occipital cortex* | 4.32 (1.36) | 4.10 (1.25) | 3.97 (0.96) | 0.56 | .57 |
| NAA CRLB |  |  |  |  |  |
| *Right inferior frontal cortex* | 2.23 (0.43) | 2.13 (0.42) | 2.00 (0.37) | 2.04 | .14 |
| *Right occipital cortex* | 2.32 (0.89) | 2.52 (0.89) | 2.30 (0.53) | 0.70 | .50 |
| % Grey matter |  |  |  |  |  |
| *Right inferior frontal cortex* | 56.65 (7.33) | 56.30 (7.35) | 54.21 (6.47) | 0.99 | .38 |
| *Right occipital cortex* | 42.91 (4.37) | 43.08 (5.54) | 43.28 (4.48) | 0.04 | .96 |
| % White matter |  |  |  |  |  |
| *Right inferior frontal cortex* | 35.37 (8.86) | 35.02 (9.20) | 38.19 (7.62) | 1.21 | .30 |
| *Right occipital cortex* | 52.80 (5.01) | 52.92 (6.85) | 53.14 (4.82) | 0.02 | .97 |
| % CSF |  |  |  |  |  |
| *Right inferior frontal cortex* | 7.19 (2.17) | 7.82 (3.08) | 6.82 (2.18) | 1.20 | .31 |
| *Right occipital cortex* | 3.94 (1.81) | 3.63 (2.02) | 3.21 (1.53) | 1.10 | .34 |

**Notes:** CRLB = Cramer-Rao lower bound, NAA = *N-*acetyl aspartate, CSF = cerebrospinal fluid.

|  | BMI Full | EDEQ  Full | BDI-II Full | BMI  HC | EDEQ  HC | BDI-II HC | BMI  BN | EDEQ  BN | BDI-II BN | BMI ANBP | EDEQ ANBP | BDI-II ANBP |
| --- | --- | --- | --- | --- | --- | --- | --- | --- | --- | --- | --- | --- |
| OCC Glu | 0.673 | 0.673 | 0.643 | 0.429 | 0.661 | 0.763 | 0.812 | 0.673 | 0.661 | 0.925 | 0.334 | 0.845 |
| OCC NAA | 0.661 | 0.429 | 0.170 | 0.661 | 0.788 | 0.362 | 0.631 | 0.631 | 0.097 | 0.788 | 0.877 | 0.925 |
| OCC Ins | 0.259 | 0.661 | 0.661 | 0.631 | 0.138 | 0.788 | 0.788 | 0.468 | 0.654 | 0.887 | 0.788 | 0.912 |
| IFC Glu | 0.877 | 0.631 | 0.776 | 0.186 | 0.661 | **0.007** | 0.643 | 0.138 | 0.877 | 0.631 | 0.776 | 0.877 |
| IFC NAA | 0.138 | 0.519 | 0.104 | 0.643 | 0.877 | 0.788 | 0.673 | 0.329 | **0.038** | 0.083 | 0.788 | 0.519 |
| IFC Ins | 0.295 | 0.259 | 0.075 | 0.362 | 0.259 | 0.673 | 0.661 | **0.038** | 0.186 | 0.643 | 0.776 | 0.097 |

**Table S2.** FDR-corrected p-values for exploratory correlation analyses

**Note:** p-values correspond to Figure S2. FDR-corrected p-values < .05 are bolded. Abbreviations: BMI = body mass index, BDI-II = Beck Depressive Inventory score, EDEQ = Eating Disorder Examination Questionnaire score, Glu = glutamate, IFC = inferior lateral prefrontal cortex, Ins = *myo*-inositol, NAA = *N*-acetyl aspartate, OCC = occipital cortex.


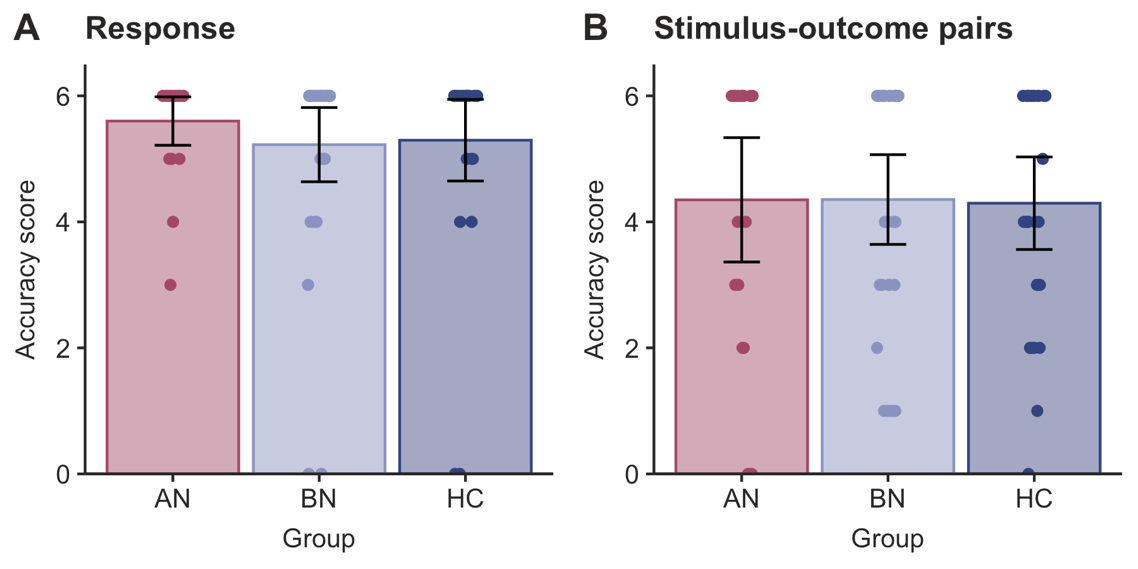


**Figure S1.** *Explicit knowledge of stimulus, response and outcome associations.* AN-BP, BN and control groups did not differ significantly in their explicit knowledge of stimulus contingencies or responses. Error bars = 95% CI.

**
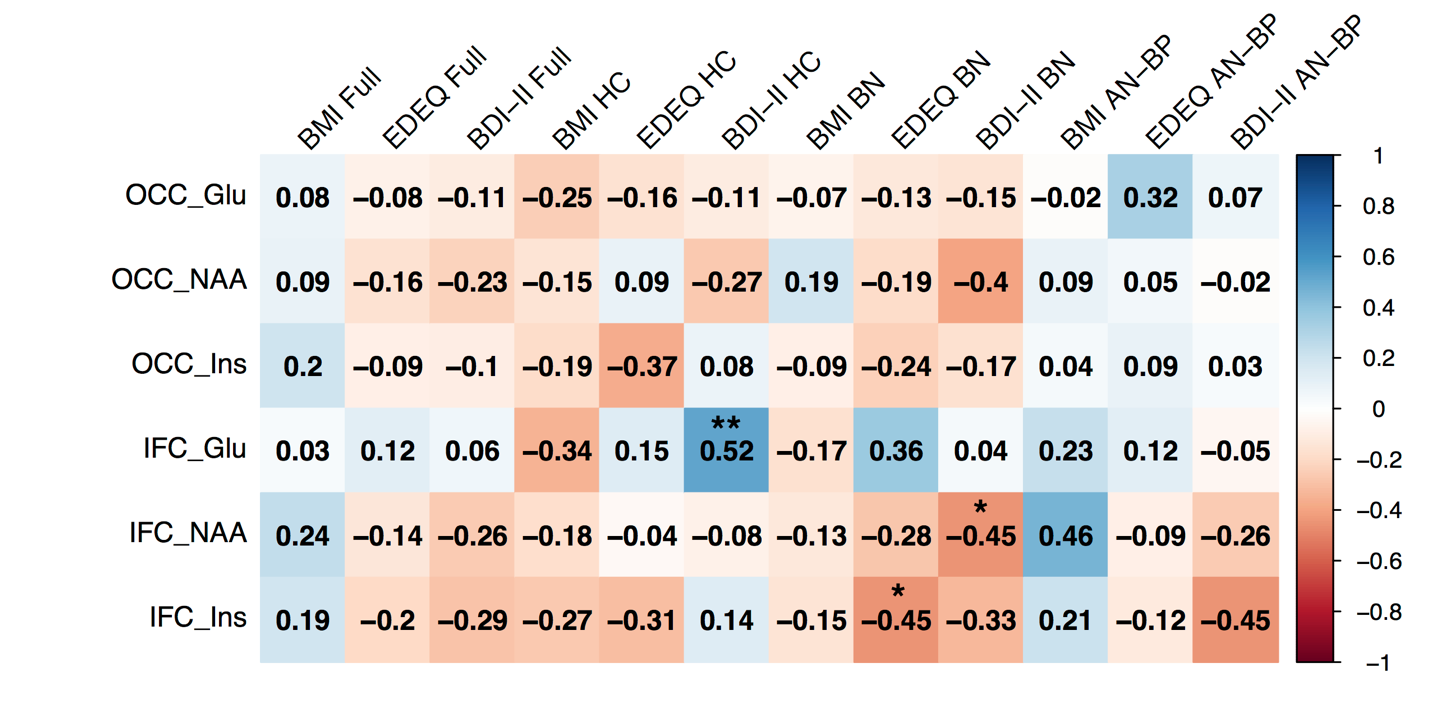
**

**Figure S2.** *Exploratory correlations between cerebral metabolites and measures of eating disorder symptom severity across participant groups.* * = q < .05, ** = q < .01. Abbreviations: BMI = body mass index, BDI-II = Beck Depressive Inventory score, EDEQ = Eating Disorder Examination Questionnaire score, Glu = glutamate, IFC = inferior lateral prefrontal cortex, Ins = *myo*-inositol, NAA = *N*-acetyl aspartate, OCC = occipital cortex.
